# The Diagnostic Dilemma of Pediatric Bilateral Proptosis in a Child With Juvenile Idiopathic Arthritis

**DOI:** 10.7759/cureus.89963

**Published:** 2025-08-13

**Authors:** Abdallah Said Abdallah, Mohamed Fadil, Siham El Haddad, Nazik Allali, Latifa Chat

**Affiliations:** 1 Department of Radiology, Children's Hospital of Rabat, Ibn Sina University Hospital Center, Mohammed V University, Rabat, MAR

**Keywords:** case report, differential diagnosis, idiopathic orbital inflammatory disease, juvenile idiopathic arthritis (jia), orbital imaging, orbital pseudotumor, pediatric proptosis, proptosis

## Abstract

Bilateral proptosis in a child presents a significant diagnostic dilemma, requiring a systematic radiological evaluation to differentiate between a spectrum of etiologies, from inflammatory conditions to malignancy. This challenge is amplified when associated with systemic autoimmune diseases, such as juvenile idiopathic arthritis (JIA). We report the case of a 12-year-old female patient with JIA who presented with bilateral proptosis ultimately diagnosed as idiopathic orbital inflammatory disease (IOID). This report describes the key imaging findings that led to the diagnosis, discusses the rarity of this clinical association, and emphasizes the pivotal role of a systematic radiological evaluation. Computed tomography (CT) and magnetic resonance imaging (MRI) were essential in characterizing the bilateral intraconal infiltrates, with the absence of bone destruction and specific signal characteristics helping to exclude key differential diagnoses. This case demonstrates that a rigorous radiological evaluation is essential for distinguishing pediatric IOID from its serious mimics and guiding appropriate patient management.

## Introduction

Idiopathic orbital inflammatory disease (IOID), also known as nonspecific orbital inflammation (NSOI) or orbital pseudotumor, is a diagnosis of exclusion characterized by a benign, non-neoplastic, non-infectious inflammatory process within the orbit for which no specific systemic or local etiology can be identified [1]. While it is the third most common orbital disorder in adults, following thyroid eye disease and lymphoproliferative disorders, its occurrence in the pediatric population is notably less frequent, accounting for approximately 6-17% of pediatric orbital inflammatory conditions [1,2].

The clinical presentation of IOID can be varied and often differs between pediatric and adult populations. In adults, IOID typically presents as an acute, unilateral, and painful process, often involving specific orbital structures like the lacrimal gland or extraocular muscles [1]. In contrast, the presentation in children is distinct; they are reported to have a higher incidence of bilateral orbital involvement and may more commonly present with systemic constitutional symptoms or associated ocular findings like iritis or optic disc edema [1-3]. The diagnostic pathway for pediatric IOID is challenging, requiring the exclusion of a broad spectrum of conditions including infections, specific inflammatory disorders (e.g., granulomatosis with polyangiitis, sarcoidosis), thyroid eye disease, vascular anomalies, and, crucially, malignancies such as rhabdomyosarcoma, lymphoma, and metastatic disease [1,4].

Imaging, particularly magnetic resonance imaging (MRI), is critical for delineating the extent and characteristics of the orbital inflammation [5]. Histopathological examination of a biopsy specimen often provides the definitive diagnosis, typically revealing a polymorphous inflammatory cell infiltrate [1,2].

We report the case of a 12-year-old female patient with juvenile idiopathic arthritis (JIA) presenting with bilateral IOID, a rare and diagnostically challenging association. This report aims to describe the key computed tomography (CT) and MRI findings and to emphasize the pivotal role of imaging in the diagnostic workup to differentiate this condition from its malignant mimics.

## Case presentation

**Figure 1:**
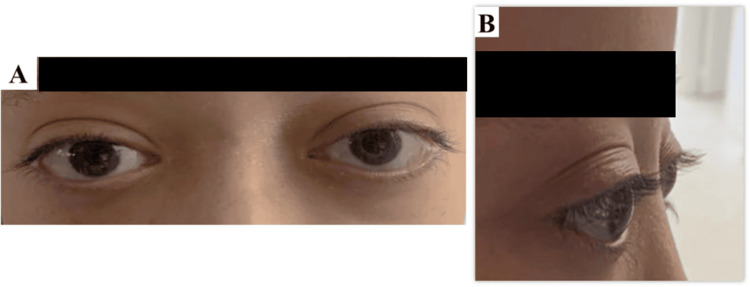
Clinical photographs of the patient at presentation Frontal (A) and right lateral (B) views showing bilateral axial proptosis, which appears more pronounced in the patient's left eye than the right. Mild bilateral upper eyelid swelling is also noted.

A 12-year-old female patient with a known history of polyarticular JIA, seronegative for both rheumatoid factor and antinuclear antibodies, presented with progressive, axial, bilateral exophthalmos. The symptoms had developed over approximately three months. Initially treated for her JIA with only non-steroidal anti-inflammatory drugs (NSAIDs), the patient's articular disease was considered quiescent at the time of presentation. The proptosis was more prominent in the left eye (Figure 1). The onset was insidious, afebrile, and painless. The exophthalmos was non-reducible, and no orbital thrill was detected.

Ophthalmological examination revealed a visual acuity of 10/10 in both eyes without correction. Ocular motility was full in all gazes, with no diplopia. Funduscopic examination and retinal angiography were unremarkable.

Laboratory investigations revealed significant systemic inflammation with associated hematological changes, as detailed in Table 1. A comprehensive workup was performed to exclude other significant pathologies. Screens for infectious diseases, including tuberculosis (Quantiferon), human immunodeficiency virus (HIV), and hepatitis B and C, were negative. Importantly, urinary catecholamine levels were also within the normal range. A bone marrow aspirate showed no evidence of malignancy. 

**Table 1:** Laboratory findings at presentation The table summarizes key laboratory results at initial presentation, highlighting severe systemic inflammation. The findings are consistent with anemia of chronic disease, leukocytosis, and thrombocytosis. Thyroid function tests were within normal limits, arguing against thyroid eye disease. CRP: C-reactive protein; ESR: erythrocyte sedimentation rate; TSH: thyroid-stimulating hormone

Investigation	Patient's value	Normal range
Inflammatory markers		
CRP	184.92 mg/L	<5 mg/L
ESR	45 mm/hour	<20 mm/hour
Complete blood count		
Hemoglobin	8.8 g/dL	12.5-16 g/dL
Leukocytes	14.65 × 10³/µL	4.0-10.0 × 10³/µL
Platelets	465 × 10³/µL	150-400 × 10³/µL
Thyroid function		
TSH	0.614 µIU/ml	0.35-4.94 µIU/ml

**Figure 2:**
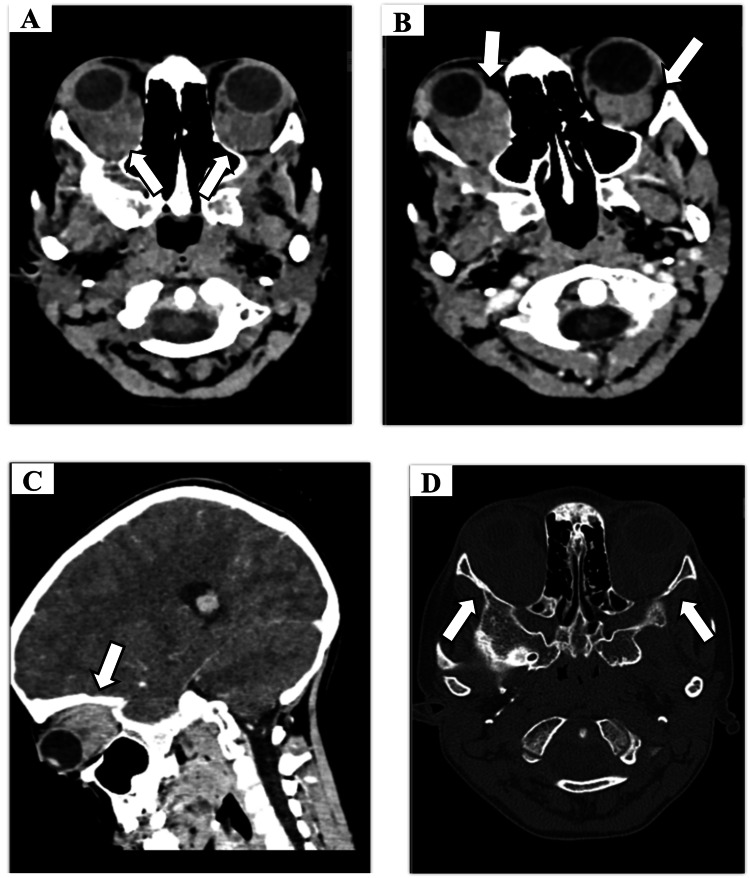
Cranio-orbital CT findings (A) Axial non-contrast CT image showing bilateral, ill-defined, isodense intraconal orbital infiltrates (arrows). (B) Axial and (C) sagittal post-contrast CT images demonstrating homogeneous enhancement of the infiltrates (arrows). (D) Axial bone window image confirming the absence of bone erosion of the orbital walls. CT: computed tomography

Orbital CT demonstrated bilateral, ill-defined, intraconal orbital infiltrates (Figure 2). These infiltrates were isodense to muscle on non-contrast sequences and exhibited homogeneous enhancement following contrast administration. No calcifications or orbital bone erosion was identified.

**Figure 3:**
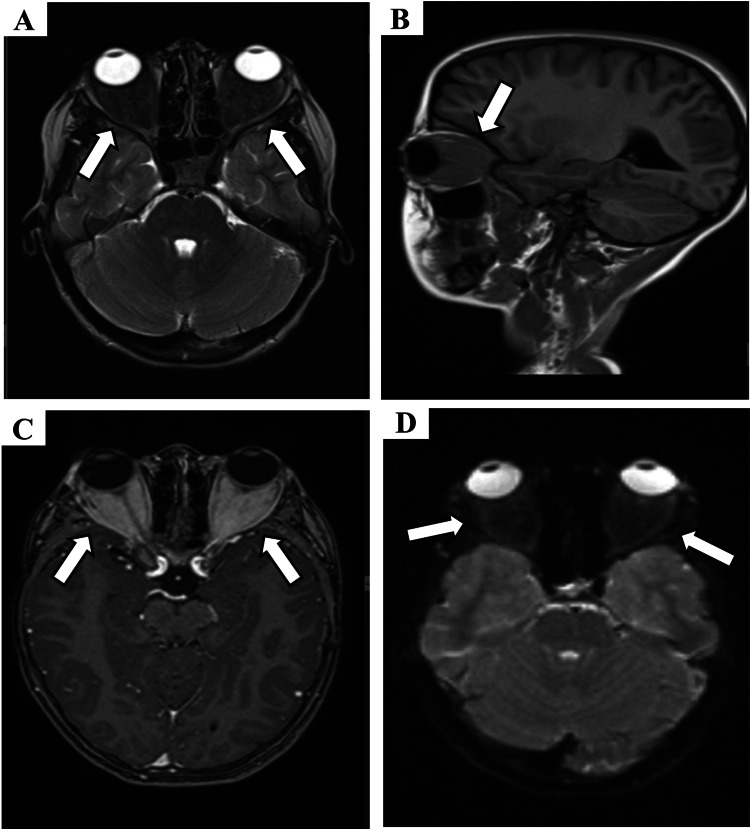
Orbital MRI characteristics Representative axial MRI sequences of the orbits. (A) T2-weighted image demonstrating bilateral, ill-defined intraconal infiltrates appearing hypointense. (B) T1-weighted image showing the infiltrates as isointense relative to extraocular muscles. (C) Post-contrast T1-weighted fat-suppressed image revealing homogeneous enhancement of the infiltrates. (D) DWI showing no restricted diffusion within the lesions. These findings are consistent with the diagnosis of idiopathic orbital inflammatory disease. MRI: magnetic resonance imaging; DWI: diffusion-weighted imaging

Subsequent MRI of the orbits further characterized these infiltrates. The lesions appeared isointense to extraocular muscles on T1-weighted images and demonstrated marked hypointensity on T2-weighted images. There was no evidence of restricted diffusion on diffusion-weighted imaging (DWI). The optic nerves were normal in appearance and caliber. The infiltrates showed significant enhancement after gadolinium administration (Figure 3).

To establish a definitive diagnosis, an orbital biopsy of the left intraconal lesion was performed via an anterior orbitotomy. Histopathological examination of the biopsy specimen revealed a reactive lymphoid infiltrate, composed of a mixed population of small mature lymphocytes, plasma cells, and occasional eosinophils, with areas of mild fibrosis. There were no features of granulomatous inflammation, vasculitis, atypical lymphoid proliferation, or malignancy.

Based on the constellation of clinical, radiological, and histopathological findings, and after the exclusion of other specific orbital pathologies, a diagnosis of IOID was made.

The patient was initiated on treatment with a bolus of intravenous methylprednisolone (Solu-Medrol®). Following this, she was transitioned to oral corticosteroids at a dose of 1 mg/kg/day (prednisolone equivalent), with a gradual tapering schedule. However, as the symptomatology did not show sufficient improvement under corticosteroid therapy alone and considering her underlying JIA, the bilateral nature of the IOID, and the desire to minimize long-term steroid exposure, therapy with methotrexate was introduced at a dose of 15 mg per week, administered orally. The patient showed a favorable clinical response to this combined regimen, with a significant reduction in proptosis and normalization of her inflammatory markers over the subsequent weeks and months. She remains under regular follow-up with both ophthalmology and rheumatology services.

## Discussion

Bilateral proptosis in a child presents a key diagnostic challenge for the radiologist, requiring a systematic approach to rule out serious pathology before a diagnosis of exclusion like IOID can be considered [1]. The association with a systemic condition such as JIA in our patient adds a layer of complexity, reinforcing the need for a meticulous workup [6].

The role of multimodality imaging is central in this process. MRI is the preferred modality for its excellent soft-tissue contrast, allowing for the detailed characterization of orbital lesions, while CT remains invaluable for assessing the bony orbit and detecting calcifications or destruction [1,5].

The primary goal of imaging is to exclude malignancy, which can mimic inflammatory conditions. Rhabdomyosarcoma, the most common primary orbital malignancy in childhood, typically presents as an enhancing extraconal mass that may cause bone erosion [4,7]. In our patient, the absence of bone destruction on CT and the intraconal location made rhabdomyosarcoma less likely. Lymphoproliferative disorders, though rare in children, are a key differential for infiltrative processes. On imaging, lymphoma often appears as a homogeneous, enhancing mass that molds to orbital structures and characteristically shows markedly restricted diffusion with low apparent diffusion coefficient (ADC) values [4,8]. Our patient's lesions did not exhibit restricted diffusion, arguing against this diagnosis. Metastatic neuroblastoma is another critical consideration, known for causing orbital bone metastases with a characteristic "hair-on-end" spiculated periosteal reaction, a feature not seen in our case [4]. Finally, optic pathway glioma, a cause of axial proptosis, was ruled out by the normal appearance and caliber of the optic nerves on MRI [4].

The association of IOID with systemic autoimmune diseases is well-established, particularly in adults with conditions like granulomatosis with polyangiitis or rheumatoid arthritis [1]. However, the link with pediatric JIA is much less frequently reported, existing primarily as isolated case reports and small series [9,10]. Published cases describe a heterogeneous spectrum of disease. The timing of presentation is variable; for instance, some reports describe orbital inflammation as the initial manifestation preceding the diagnosis of JIA [9], while others document atypical presentations occurring concurrently with the systemic illness [10]. Our case contributes to the limited literature on the rare association between JIA and IOID and reinforces the need to consider IOID in the differential diagnosis of orbital proptosis in this patient population, even in the absence of active arthritis.

Once malignancy is reasonably excluded, common inflammatory and vascular conditions must be considered. Thyroid eye disease is the most frequent cause of bilateral proptosis in children, but its hallmark radiological sign is the enlargement of extraocular muscle bellies with characteristic sparing of the tendons [1,11]. This was contrary to our patient's findings where the process was an intraconal infiltrate not centered on the muscles. Orbital cellulitis, the main clinical mimic, is typically associated with sinus disease and can lead to abscess formation, seen as a ring-enhancing collection on imaging, which was absent in our case [11].

Other less common pediatric entities are also part of the differential. Langerhans cell histiocytosis (LCH) classically presents as a "punched-out" lytic lesion of the orbital bones [4]. Ruptured dermoid cysts can incite intense inflammation but often contain lipid-density material on CT or show fat signal on MRI, findings not present in our patient [1]. Systemic inflammatory conditions such as sarcoidosis or granulomatosis with polyangiitis can also present with orbital masses and must be considered, especially in bilateral cases [6,12].

This systematic exclusion leads to the diagnosis of IOID. The imaging findings in our case, bilateral, ill-defined, enhancing intraconal infiltrates demonstrating T1 isointensity and marked T2 hypointensity, are highly characteristic of the fibro-inflammatory nature of some forms of IOID [1,4]. The T2 hypointensity, in particular, suggests a less edematous, more cellular or fibrotic process, consistent with the biopsy results. The diagnosis was therefore established not only by the presence of these suggestive features but also by the crucial absence of signs pointing to its mimics.

Despite the power of advanced imaging, its limits must be acknowledged. A significant overlap exists between the appearance of IOID and lymphoma, and in atypical cases, a definitive diagnosis cannot be made on imaging alone [4,8]. Therefore, as was done in our case, histopathological confirmation via biopsy remains the gold standard to definitively rule out malignancy before committing a child to long-term immunosuppressive therapy [13]. Once the diagnosis of IOID was confirmed, appropriate management could be initiated, leading to a favorable outcome [14].

## Conclusions

This case of bilateral proptosis in a child with JIA highlights a significant diagnostic challenge. A systematic radiological approach is essential to characterize the orbital lesions and rule out serious mimics like malignancy. Our report adds to the limited literature on the rare association between JIA and IOID. Ultimately, this case underscores the pivotal role of radiology in navigating a complex differential diagnosis to ensure timely and appropriate management.
